# Superior *in vivo* Wound-Healing Activity of Mycosynthesized Silver Nanogel on Different Wound Models in Rat

**DOI:** 10.3389/fmicb.2022.881404

**Published:** 2022-06-02

**Authors:** Swapnil Gaikwad, Sonal Birla, Avinash P. Ingle, Aniket Gade, Pramod Ingle, Patrycja Golińska, Mahendra Rai

**Affiliations:** ^1^Department of Biotechnology, SGB Amravati University, Amravati, India; ^2^Microbial Diversity Research Center, Dr. D. Y. Patil Biotechnology and Bioinformatics Institute, Dr. D. Y. Patil Vidyapeeth, Pune, India; ^3^Biotechnology Centre, Department of Agricultural Botany, Dr. Panjabrao Deshmukh Agricultural University, Akola, India; ^4^Department of Microbiology, Nicolaus Copernicus University, Torun, Poland

**Keywords:** wound healing, bio-AgNPs, silver nano-gel, mycosynthesis, albino Wistar rat, histology

## Abstract

Wound healing is a complex phenomenon particularly owing to the rise in antimicrobial resistance. This has attracted the attention of the scientific community to search for new alternative solutions. Among these, silver being antimicrobial has been used since ancient times. Considering this fact, the main goal of our study was to evaluate the wound-healing ability of mycofabricated silver nanoparticles (AgNPs). We have focused on the formulation of silver nanogel for the management of wounds in albino Wistar rats. Mycosynthesized AgNPs from *Fusarium oxysporum* were used for the development of novel wound-healing antimicrobial silver nanogel with different concentrations of AgNPs, i.e., 0.1, 0.5, and 1 mg g^−1^. The formulated silver nanogel demonstrated excellent wound-healing activity in the incision, excision, and burn wound-healing model. In the incision wound-healing model, silver nanogel at a concentration of 0.5 mg g^−1^ exhibited superior wound-healing effect, whereas in the case of excision and burn wound-healing model, silver nanogel at the concentrations of 0.1 and 1 mg g^−1^ showed enhanced wound-healing effect, respectively. Moreover, silver nanogel competently arrests the bacterial growth on the wound surface and offers an improved local environment for scald wound healing. Histological studies of healed tissues and organs of the rat stated that AgNPs at less concentration (1 mg g^−1^) do not show any toxic or adverse effect on the body and promote wound healing of animal tissue. Based on these studies, we concluded that the silver nanogel prepared from mycosynthesized AgNPs can be used as a promising antimicrobial wound dressing.

## Introduction

The skin is the main structure of the body that acts as a barrier against foreign agents. Loss of skin tissue integrity results in wounds or infections that could be fatal. The wound interrupts physiological functions by interfering with the cellular and anatomic stability of tissues. Various agents, such as physical, chemical, or microbial, may be responsible for wound formations, which are unavoidable events of life. Therefore, wound healing is a complex and main biological response that helps in the renewal of tissue integrity and body functions, which includes an integrated cascade of continuous and overlapping biological events (Rajakumari, 2019; Wilkinson and Hardman, 2020). Wound healing involves the systematic progress of events that regenerate the integrity of the damaged tissue (Sorg et al., 2017).

Wound healing involves three main stages, namely, inflammatory, proliferative, and remodeling phases. An important point in wound healing is to heal the wound in a short time, with negligible pain, discomfort, and blemishing to the patient. It must occur in a physiological state favorable to tissue repair and regeneration (Wallace et al., 2021). Until the injured surfaces are firmly joined by collagen, the healing process is incomplete (Buffoni et al., 1993; Rodrigues et al., 2019). Later, wound healing results in bacterial infections, stress, and nutritional deficiencies due to which the wound-healing materials with antimicrobial properties are in high demand. To minimize tissue injury and offer adequate tissue perfusion and oxygenation, appropriate nutrition, and moisture are the basic principles of optimal wound healing (Mir et al., 2018; Shi et al., 2020). After an injury, an inflammatory response occurs and the cells under the dermis initiate to increase collagen formation (Xue and Jackson, 2015) and, after that, regeneration of epithelial tissue starts. Other than AgNPs, iron oxide nanoparticles (NPs) have been shown to possess anticancer, antioxidant, antibacterial, and anti-scratch activity, making them compatibly used in wound dressing materials and antibiotic substitutes (Majeed et al., 2021).

Silver nitrate is a well-known antimicrobial agent used in the treatment of chronic wounds and for many other medical purposes since ancient times (Paladini and Pollini, 2019; Talapko et al., 2020; Chinnasamy et al., 2021). Silver NPs (AgNPs) show a potent activity against a broad range of Gram-negative and Gram-positive, aerobic and anaerobic bacteria, filamentous fungi, yeast, and viruses (Gaikwad et al., 2013a; Dakal et al., 2016; Alavi and Rai, 2021; Lotfy et al., 2021; Zawadzka et al., 2021; Alavi et al., 2022). In the beginning of the twentieth century, morbidity, and mortality were major consequences of burn wound infections (Norbury et al., 2016; Ladhani et al., 2021). After burn injury, normal skin barrier and many of the systemic host defense mechanisms get interrupted, making skin susceptible to microbial infections and ultimately resulting in the development of sepsis. Due to such reasons, antimicrobial agents containing silver (e.g., silver nitrate and silver sulfadiazine) have modernized burn wound care and considerably reduced morbidity and mortality (Minasyan, 2019; Ueda et al., 2020).

Colloidal silver for wound treatment was approved by the US Food and Drug Administration in 1920 (Sim et al., 2018). In the 1940s, after the arrival of antibiotics, the medical application of silver declined dramatically. After 24 years, Moyer et al. (1965) relighted the research interest in silver by using a 0.5% silver nitrate solution in the burn area.

To target the various phases of wound healing, different drugs and delivery systems have been widely studied. Still, the problem persists with currently available wound treatments, which include repeated dressing, and wounds have more risk of infections until it heals completely. Especially in chronic wounds where there is poor blood circulation and local edema, healing of wounds is very difficult without any active treatment. Moreover, the present treatments are costly and less effective, which encouraged the development of novel therapeutics (Frykberg and Banks, 2015; Saghazadeh et al., 2018).

At present, a number of NPs have been used in wound healing, such as polylactic-co-glycolic acid (PLGA)-curcumin NPs (Mobaraki et al., 2021), mucoadhesive NPs with enoxaparin (Yan et al., 2020), calcium-based NPs (Subramaniam et al., 2021), magnetic NPs (Noh et al., 2019), titanium dioxide NPs (Nikpasand and Parvizi, 2019), and copper NPs (Alizadeh et al., 2019).

Biological dressings, namely, chitosan films (Cunha et al., 2020) and the application of AgNPs, are well-known for faster wound healing (Durán et al., 2007; Paladini and Pollini, 2019; Nqakala et al., 2021). The chitosan, cellulose, and AgNPs, when used in combination, showed better activity, as compared to those used alone. Ahamed and Sastry (2011) evaluated the combined film activity of chitosan–cellulose–AgNPs for antibacterial activity, absorption capacity, tensile strength, and *in vivo* wound-healing activity by the excision wound model using albino rats. Sundaramoorthi et al. (2009) studied the activity of AgNPs synthesized from *Aspergillus niger* in excision and thermal wound model.

Wu et al. (2014) synthesized and self-assembled AgNPs on the surface of bacterial cellulose (BC) nanofibers and studied its antibacterial activity, cytocompatibility, and wound-healing effect. Modified cationic biopolymer, guar gum alkylamine (GGAA), impregnated with biologically synthesized AgNPs was used as a complex in the rodent wound-healing model (Ghosh Auddy et al., 2013). They found that this complex showed better healing and enhanced cosmetic appearance as compared to commercially available silver alginate cream. Krishnan et al. (2020) explored the wound-healing characteristics of AgNPs in an animal model and concluded the fast healing and improved cosmetic appearance in a dose-dependent manner. In addition, they investigated the quantitative PCR, immunohistochemistry, and proteomic studies which revealed that AgNPs exhibited positive effects through their antimicrobial properties, decrease in wound inflammation, and modulation of fibrogenic cytokines (Ahmed et al., 2022).

Hendi (2011) studied the effect of AgNPs on the liver and kidney, while wound healing showed modulation in liver and kidney functions. Ultrafine gelatin fiber-containing AgNPs exhibited antimicrobial activity against some bacteria found in burn wounds, i.e., *Pseudomonas aeruginosa, Staphylococcus aureus, Escherichia coli*, and methicillin-resistant *S. aureus*. Majeed et al. (2014) reported the *Penicillium* sp. biomass-mediated synthesis of AgNPs with a size of 20–45 nm having an activity against bacterial pathogens and activator for amoxicillin. Other *Penicillium* spp. were reported to have the ability of intracellular and extracellular synthesis of AgNPs with inhibitory and bactericidal activities. These *Penicillium-*mediated AgNPs were tested against *Bacillus cereus, Proteus vulgaris, S. aureus*, and *Staphylococcus epidermidis*. The results showed the potential use of AgNPs against common human pathogens and substitute high-dose antibiotics (Ingle et al., 2022).

Silver treatment has many advantages, e.g., silver reduces the chances of developing resistant bacteria, has efficiency against multidrug-resistant organisms, and has low systemic toxicity. Although silver compounds are used topically, silver nitrate and silver sulfadiazine have some disadvantages. It could get neutralized by anions (bicarbonate, chloride, and protein) in body fluids or cause cosmetic aberration, namely, argyria (blue-gray coloration) in long-term use, or could stop the healing process due to fibroblast and epithelial cell toxicity.

In this study, we provided an alternative silver delivery treatment for skin wounds with AgNPs synthesized in the previous study (Gaikwad et al., 2013b). Extracellular bioengineered AgNPs under optimized conditions can be used as a potent weapon against multidrug-resistant bacterial pathogens. The toxicity effects of AgNPs against bacterial pathogens have been demonstrated by testing them against various Gram-positive and Gram-negative pathogens (Nanda et al., 2017; Muthukrishnan et al., 2019). We formulated AgNPs containing gel for the management of excision, incision, and burn wounds in albino Wistar rats. In addition, the histopathological studies of skin and organs have been studied to evaluate toxicological parameters.

## Materials and Methods

### Mycosynthesis and Characterization of AgNPs

The AgNPs were synthesized using the fungus *Fusarium oxysporum* (Ghosh Auddy et al., 2013). The synthesis of AgNPs was preliminarily confirmed by UV-Vis spectroscopy which gave a characteristic peak for NPs. Further confirmation and characterization of AgNPs were carried out using Nanoparticle Tracking Analysis (LM 20), Zeta potential measurement, and transmission electron microscopy (TEM) analysis.

### Purification and Powder Preparation of AgNPs

The synthesized colloidal AgNPs were passed through a membrane filter of size 0.2 μm to remove impurities and fungal spores. Then, these NPs were concentrated by repeated centrifugation at 20,000 rpm for 30 min at 4°C. The concentrated NPs were dried to form nano-powder which was then used to prepare stocks of different known concentrations.

### Formulation of Silver Nanogel

Silver nanogels having different concentrations of AgNPs, i.e., 0.1, 0.5, and 1 mg g^−1^ were prepared using Carbopol (Ultrez 20). In all formulation, 1.5% (w/v) Carbopol was sprinkled slowly in sterilized distilled water containing AgNPs with stirring to disperse it properly. Glycerin, propylene glycol, and triethanolamine were also added one by one into the main mixing vessel with stirring.

### Evaluation of Silver Nanogel for Wound-Healing Property

Albino Wistar rats (male) were procured from the National Institute of Biosciences, Pune (MS).

All the procedures for animal experiments were approved by the Institutional Animal Ethics Committee (Vide letter No. GCOPA/IAEC/2012/404, Institutional Animal Ethics Committee Reg. No. 1370/ac/10/CPCSEA).

Three types of model (wound) were studied for wound-healing activity on albino Wistar rats.

### Incision Wound Healing: To Study the Tensile Strength of Wounds

To study the tensile strength of the wound, albino Wistar rats weighing 150–200 g were divided into six groups with six rats in each group. Para-vertebral straight incision of 4–5 cm was made on anesthetized rat at the shaved back side of the neck. After complete hemostasis, the wounds were closed by suture placed at ~1 cm apart, and each group was treated daily with their respective formulations for 9 days. Each rat was provided with a separate cage, food, and water. Group A was treated with gel with AgNPs at a concentration of 0.1 mg g^−1^, Group B was treated with gel with AgNPs at a concentration of 0.5 mg g^−1^, Group C was treated with gel with AgNPs at a concentration of 1 mg g^−1^, Group D was treated with market drug silverex^®^ 0.2% (positive control), Group E was treated with only gel base (gel without any drug), and Group F was kept as untreated (negative control). Sutures were removed on the ninth day and tensile strength was measured on the 10th day by applying weights.

### Excision Wound-Healing Model

In the study of excision wound healing also, animals were divided into six groups of six rats in each group. Excision wounds were inflicted on anesthetized rats by cutting away 300 mm^2^ on depilated and marked back of each rat. The wounds were treated the same as in the incision wound-healing model for 20 days. A wound contraction rate at the interval of 5 days for each animal of respective groups was measured by tracing the wound on graph paper. Reduction in the wound area was expressed as percentage contraction of the original wound calculated by the following formula:


$$\text{Percentage of wound concentration }=\;\frac{\text{initial}\;\text{day}\;\text{wound}\;\text{size}-\text{specific}\;\text{day}\;\text{wouund}\;\text{size}}{\text{initial}\;\text{day}\;\text{wound}\;\text{size}}\text{ x100}$$

### Burn Wound-Healing Model

Burn wounds were inflicted on each anesthetized rat by pouring hot molten wax at 80°C into a metal cylinder of 300 mm^2^ of circular opening kept at shaved back of the rat. Burn wounds were treated by drug of respective groups same as in the excision wound-healing model for 15 days. A wound contraction rate at the interval of 5 days for each animal of respective groups was measured by tracing the wound on graph paper. Reduction in the wound area was calculated as a percentage contraction of the original wound.

### Histology of Animal Tissue (Healed Area) and Organs

After complete healing of wound tissues removed from the wound bed and after gross necropsy, organs of the rats, i.e., brain, heart, kidney, liver, and lung were removed and preserved in 10% neutral buffered formalin till the histological study, before the staining process tissues were dehydrated by graded series of alcohols. Later, tissue was treated with xylene and fixed in paraffin. Sections were cut from tissues of respective groups and stained with hematoxylin & eosin for microscopic examination. Furthermore, the slides were mounted with DPX and observed for collagen content, inflammatory changes, vacuolization, fibroblast, and polymorphonuclear cells.

## Results

**Figure 1:**
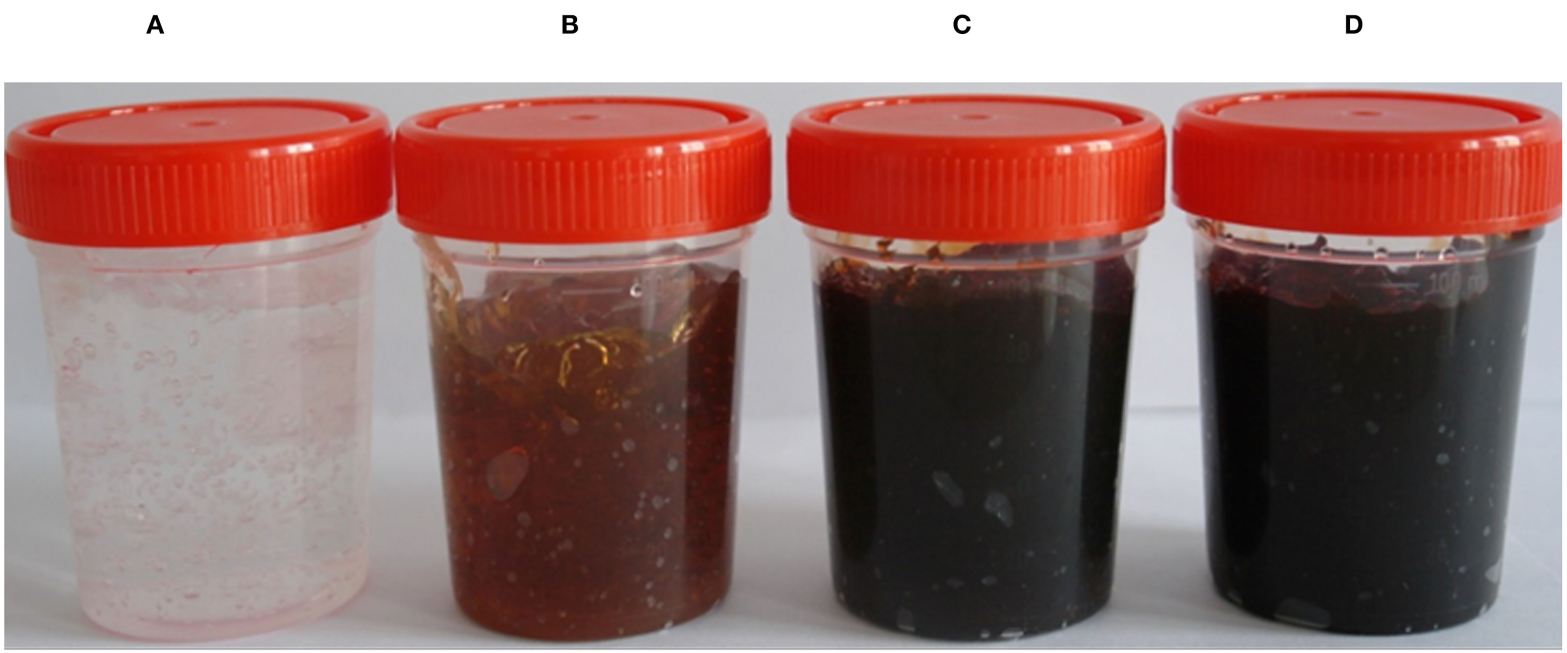
Formulated silver nanogels of different concentrations. **(A)** Control (Gel base); **(B)** 0.1 mg g^−1^; **(C)** 0.5 mg g^−1^; **(D)** 1 mg g^−1^ (mg g^−1^ concentration of silver NPs used for gel preparation), (Patented product, Patent No. 377757, Application No. 838/MUM/2014).

Silver nanogels having different concentrations of AgNPs, i.e., 0.1, 0.5, and 1 mg g^−1^ (Figure 1) were prepared, and their wound-healing activity was evaluated. The pH of the formulated silver nanogel was maintained in the range of 6–7.

The study has been carried out on two models, such as incision wound-healing model and excision wound-healing model. In the case of the incision wound-healing model, formulated nanogel, marketed drug, and gel base were applied; however, one group was kept untreated for 9 days. After removing the sutures of the wound, on the 10th day, tensile strength was measured by applying the weight to evaluate the degree of wound healing.

**Figure 2:**
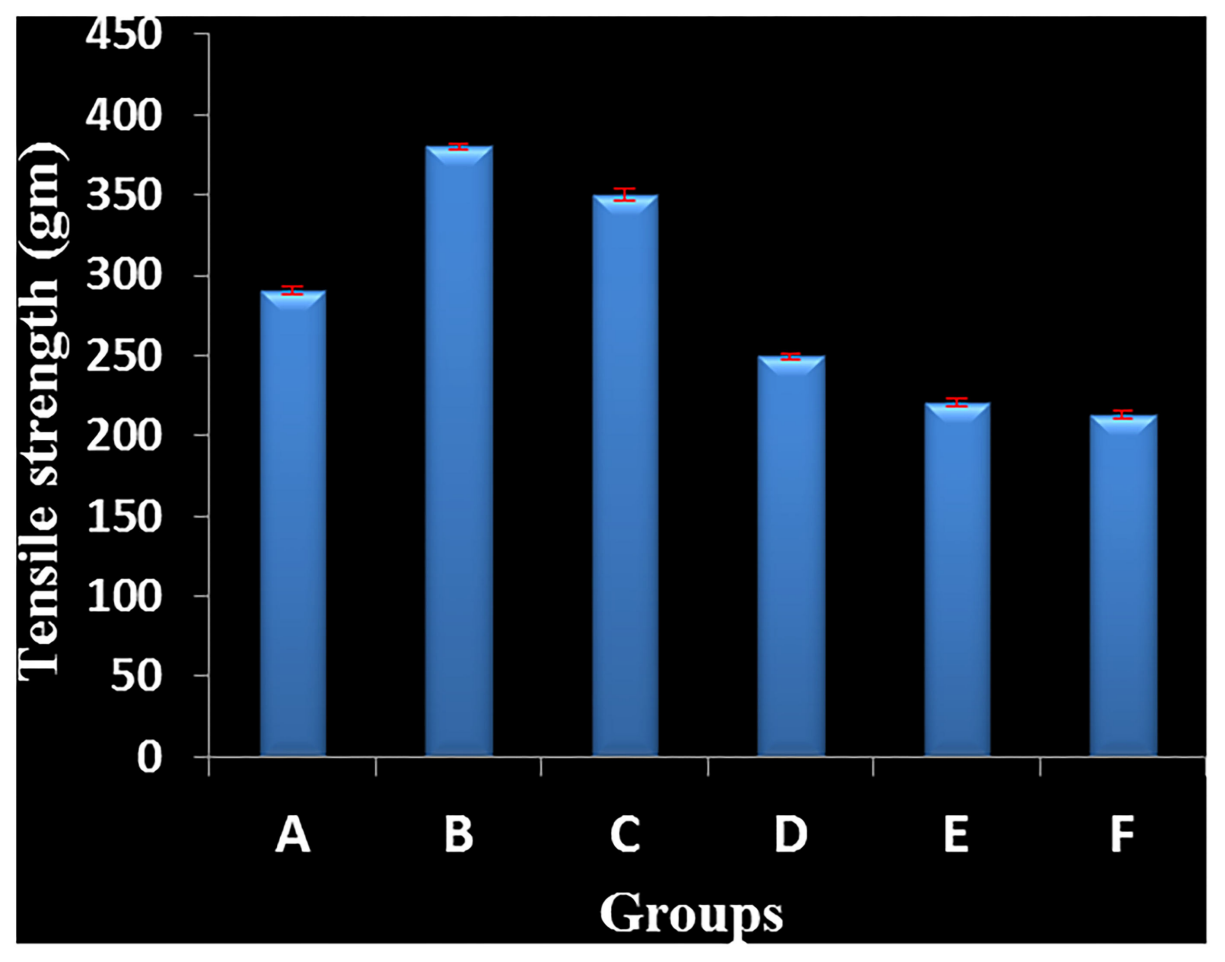
Effect of silver nanogel on incision wound model (tensile strength) in albino Wistar rats. Group A: Test treatment group (silver nanoparticles (AgNPs) 0.1 mg g^−1^). Group B: Test treatment group (AgNPs 0.5 mg g^−1^). Group C: Test treatment group (AgNPs 1 mg g^−1^). Group D: Positive control (market drug silverex 0.2%). Group E: Group treated with gel base. Group F: Negative control (untreated group).

The wound treated with gel base, market drug (silverex 0.2%), and control (without any treatment) showed a less tensile strength of 210, 250, and 220 g, respectively. In contrast, formulated nanogels of three different concentrations of 0.1, 0.5, and 1 mg g^−1^ showed remarkable tensile strength, i.e., 290, 380, and 350 g, respectively (Table 1). It clearly indicates that wound-healing agents promote a gain in tensile strength. Even in comparison with market drug, nanogels having a 0.5 mg g^−1^ concentration of AgNPs showed the superior tensile strength in wound healing, i.e., 380 ± 5 g (Figure 2).

**Table 1:** Effect of silver nanogel on incision wound model (tensile strength) in albino Wistar rats (expressed in mean ± SD).

**Groups**	**Tensile strength (g)**
Group A	291 ± 2.64
Group B	380.33 ± 2.0
Group C	350 ± 4.0
Group D	249.33 ± 2.08
Group E	220.33 ± 2.51
Group F	213 ± 2.64

*Where, Group A: Test treatment group (AgNPs 0.1 mg g^−1^). Group B: Test treatment group (AgNPs 0.5 mg g^−1^). Group C: Test treatment group (AgNPs 1 mg g^−1^). Group D: Positive control (market drug silverex 0.2%). Group E: Group treated with gel base. Group F: Negative control (untreated group)*.

**Figure 3:**
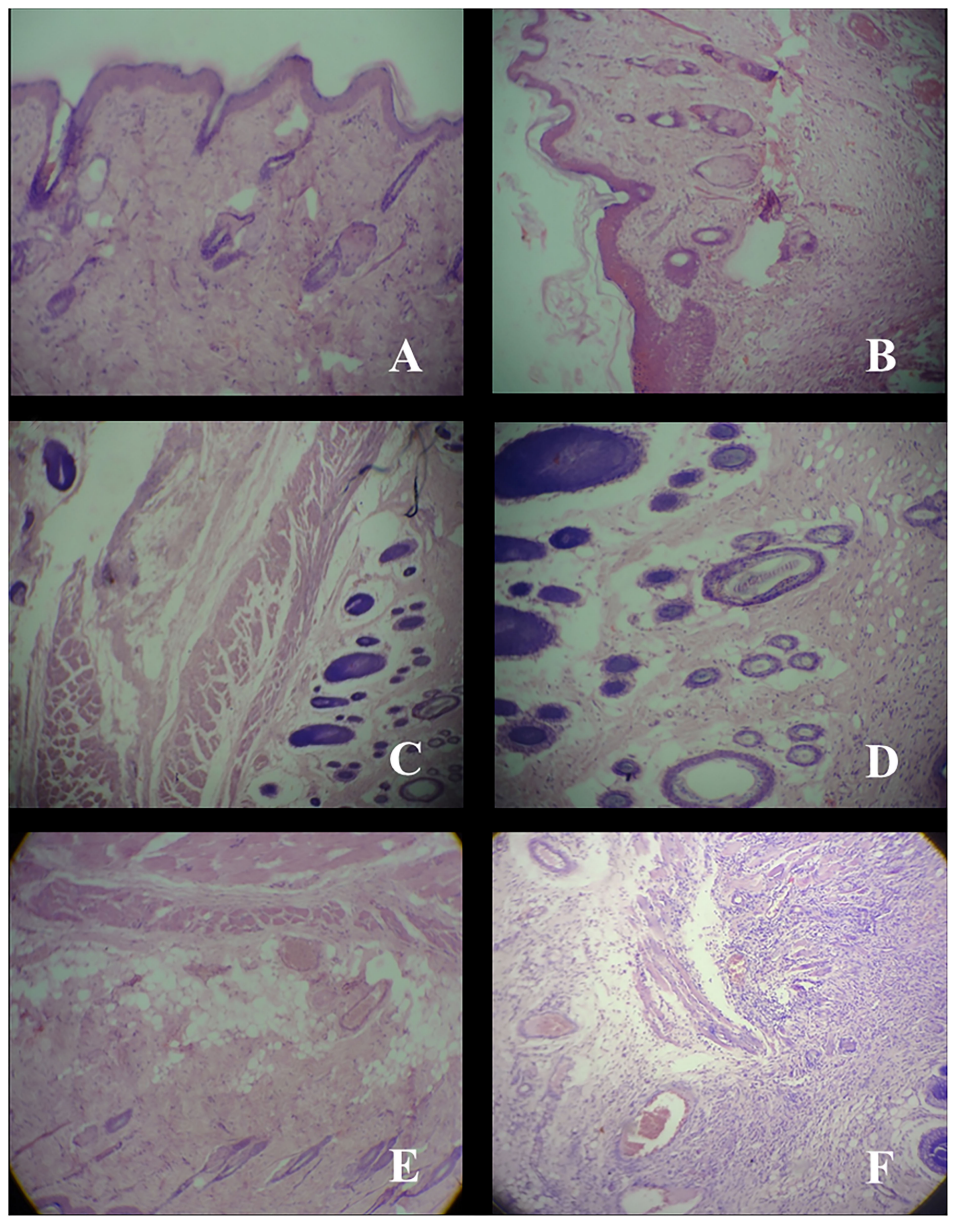
Histology of skin tissue taken from healed area after wound-healing study at a resolution of 40×. **(A)** Group A; Thin epiderm, no inflammatory. **(B)** Group B; Thin epiderm, collagen. **(C)** Group C; Thick collagen, regenerating hair follicle. **(D)** Group D; Cellular infiltration, regenerating hair follicle. **(E)** Group E; Edema, congestion, persistent inflammatory cell. **(F)** Group F; Congestion, inflammatory cell.

After complete wound healing, tissues were taken from healed area for histological study. In Group A (mice treated with nanogels with 0.1 mg g^−1^ AgNPs), thin epidermis and no inflammatory cells were found (Figure 3A). In Group B (mice treated with nanogels with 0.5 mg g^−1^ AgNPs), thin epidermis with thin collagen was observed (Figure 3B). However, in Group C (mice treated with nanogels with 1 mg g^−1^ AgNPs), thick collagen and regenerating hair follicles were observed (Figure 3C). In Group D (mice treated with silverex 0.2%) also, regenerating hair follicles were observed but with cellular infiltration (Figure 3D). In contrast, in control, i.e., Group E (gel base only) (Figure 3E) and group F (untreated) (Figure 3F), edema, congestion, and inflammatory cells were observed. Silver nanogel at the concentrations of 0.5 and 1 mg g^−1^ showed high collagen content and regenerating hair follicles as compared to silver nanogel at a concentration of 0.1 mg g^−1^.

**Figure 4:**
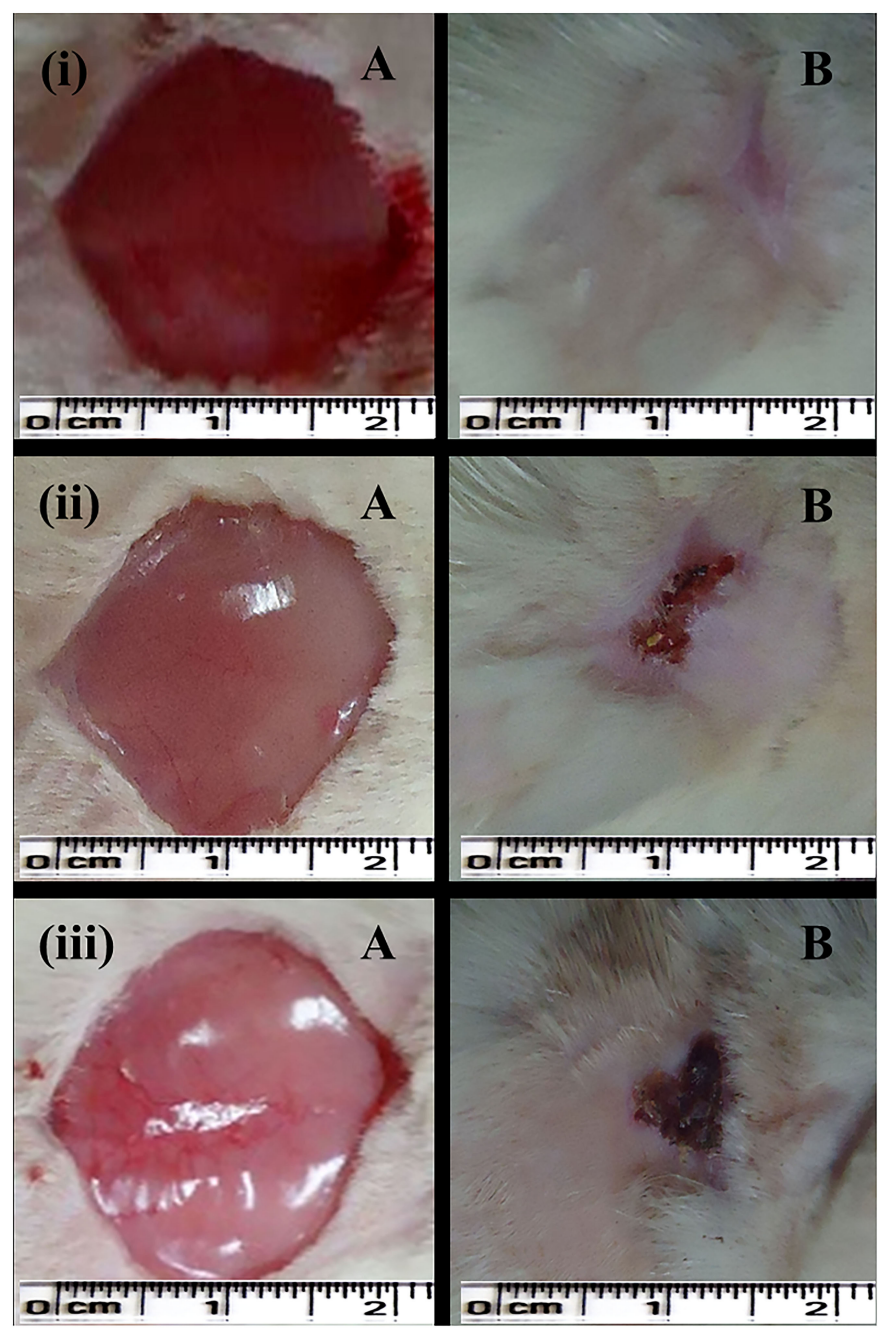
Excision wound-healing model (albino Wistar) for formulated silver nanogel, marketed drug, and untreated (control) group. (i) Excision wound treated with silver nanogel at a concentration of 0.1 mg g^−1^ (Group A); (ii) Excision wound treated with marketed drug silverex 0.2% (Group D); (iii) Untreated (control) group (Group F), where **(A)** Excision wound created on the first day and **(B)** Excision wound completely healed on 20th day after treatment.

In the case of the excision wound-healing model, wounds were inflicted by cutting away 300 mm^2^ on the back of the rat and then a drug of interest and control were applied twice a day for up to 20 days (Figure 4). A wound contraction for each animal with respective groups was observed and traced after every 5 days on graph paper. After 20 days, the control group (without any treatment) and only gel base showed less wound contraction, i.e., 85.77 ± 7.45% and 87.27 ± 3.64%, respectively (Table 2).

**Table 2:** Effect of silver nanogel on excision wound model (percentage wound contraction) in albino Wistar rats.

**Groups**	**Days**			
	**5th (% contraction)**	**10th (% contraction)**	**15th (% contraction)**	**20th (% contraction)**
Group A	33.22 ± 8.88	68.76 ± 21.95	87.14 ± 2.74	**98.30 ±1.43**
Group B	45.96 ± 8.66	69.10 ± 8.59	79.11 ± 10.59	**94.23 ±6.65**
Group C	38.06 ± 12.50	67.16 ± 17.81	79.88 ± 12.54	91.79 ± 10.77
Group D	29.70 ± 8.07	61.26 ± 11.18	80.38 ± 3.20	93.19 ± 4.53
Group E	14.32 ± 8.80	49.71 ± 15.83	84.76 ± 2.04	87.27 ± 3.64
Group F	27.42 ± 14.11	54.84 ± 8.87	80.31 ± 4.78	85.77 ± 7.45

*Values (expressed in mean ± SD) tensile strength. Where, Group A: Test treatment group (Silver nanoparticles 0.1 mg g^−1^). Group B: Test treatment group (Silver nanoparticles 0.5 mg g^−1^). Group C: Test treatment group (Silver nanoparticles 1 mg g^−1^). Group D: Positive control (market drug silverex 0.2%). Group E: Group treated with gel base. Group F: Negative control (untreated group)*.

*Bold values are of maximum contraction of wound as compared to other treatments.*

Nanogel with AgNPs at a concentration of 0.1 mg g^−1^ showed a remarkable wound contraction, i.e., 98.30 ± 1.43% (Figure 4); in contrast, nanogel with AgNPs at the concentrations of 0.5 and 1 mg g^−1^ revealed a wound contraction of 94.23 ± 6.65 and 91.79 ± 10.77%, respectively. Group C having the AgNPs at a concentration of 1 mg g^−1^ showed a comparatively less wound contraction. Moreover, the percentage of wound contraction was significant on the 15th and 20th days in all the formulated nanogel as compared to control groups. Moreover, delayed eschar formation was observed in control groups. Results have been expressed in mean ± SD, and were analyzed by one-way analysis of variance (ANOVA) (Table 3).

**Table 3:** ANOVA for the effect of silver nanogel on excision wound model in albino Wistar rats.

**Source**	**SS**	**Df**	**MS**	***F***
Columns	13012.3	3	4337.45	133.89
Rows	530	5	106.01	3.27
Error	485.9	15	32.4	
Total	14028.3	23		
Source	SS	df	MS	F

*P < 0.05, significant in comparison with control*.

**Figure 5:**
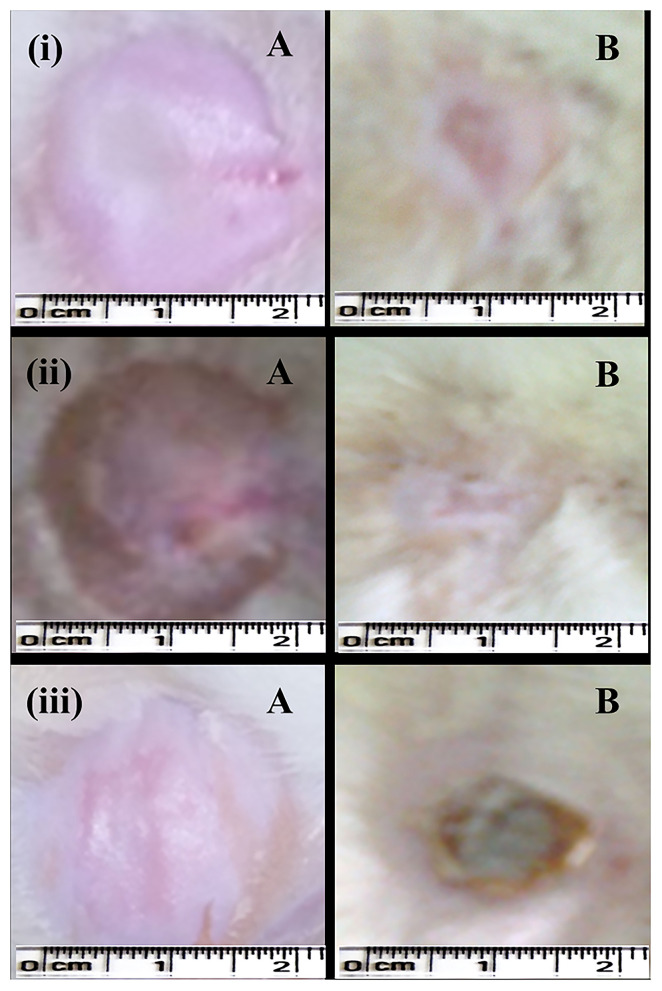
Burn wound-healing model (albino Wistar) for formulated silver nanogel, marketed drug, and untreated (control) group. (i) Burn wound treated with silver nanogel at a concentration of 1 mg g^−1^ (Group C); (ii) Burn wound treated with marketed drug silverex 0.2% (Group D); (iii) Untreated (control) group (Group F), where **(A)** Burn wound created on the first day and **(B)** Burn wound healed on 15th day after treatment.

As silver has a crucial role in the healing of thermal wounds, we also studied the burn wound-healing model for formulated silver nanogel. After inflicting thermal wounds on the backs of animals, formulated nanogel and control drug were applied twice a day (Figure 5). The same as in the excision wound model contraction of the wound is traced after every 5 days for each group on graph paper. Market drug (silverex 0.2%) showed the better wound contraction, i.e., 99.65 ± 0.60%.

Among the formulated nanogel, 1 mg g^−1^ AgNPs showed a superior wound contraction, i.e., 98.60 ± 2.41% (Figure 5) followed by a concentration of 0.5 mg g^−1^ that showed 96.42 ± 3.31% contraction. The percentage of wound contraction in nanogel and silver nitrate (silverex 0.2%) is significant after 10th day as compared to control (Table 4). Results of the burn wound-healing model were expressed in mean ± SD and were analyzed by ANOVA (Table 5). The epithelization period was faster in the experimental group, which was monitored by noting the number of days required for the scar to fall off from the burn wound surface without leaving a raw wound behind.

**Table 4:** Effect of silver nanogel on burn wound model (percentage wound contraction) in albino Wistar rats.

**Groups**	**Days**		
	**5th** **(% contraction)**	**10th** **(% contraction)**	**15th** **(% contraction)**
Group A	33.61 ± 16.80	60.61 ± 5.82	92.79 ± 0.68
Group B	65.76 ± 30.15	82.13 ± 15.48	**96.42 ±3.31**
Group C	75.35 ± 21.69	82.07 ± 15.87	**98.60 ±2.41**
Group D	68.81 ± 27.02	81.23 ± 16.26	99.65 ± 0.60
Group E	69.24 ± 27.52	82.51 ± 15.36	87.01 ± 4.73
Group F	63.36 ± 31.78	68.59 ± 28.32	85.48 ± 15.99

*Values (expressed in mean ± SD) wound contraction %. Where, Group A: Test treatment group (Silver nanoparticles 0.1 mg g^−1^). Group B: Test treatment group (Silver nanoparticles 0.5 mg g^−1^). Group C: Test treatment group (Silver nanoparticles 1 mg g^−1^). Group D: Positive control (market drug silverex 0.2%). Group E: Group treated with gel base. Group F: Negative control (untreated group)*.

*Bold values are of maximum contraction of wound as compared to other treatments*.

**Table 5:** ANOVA for the effect of silver nanogel on burn wound model in albino Wistar rats.

**Source**	**SS**	***df***	**MS**	***F***	**Prob>F**
Columns	3090.77	2	1545.39	30.74	0.0001
Rows	1160.5	5	232.1	4.62	0.0192
Error	502.76	10	50.28		
Total	4754.03	17			

*P < 0.05, significant in comparison with control*.

**Figure 6:**
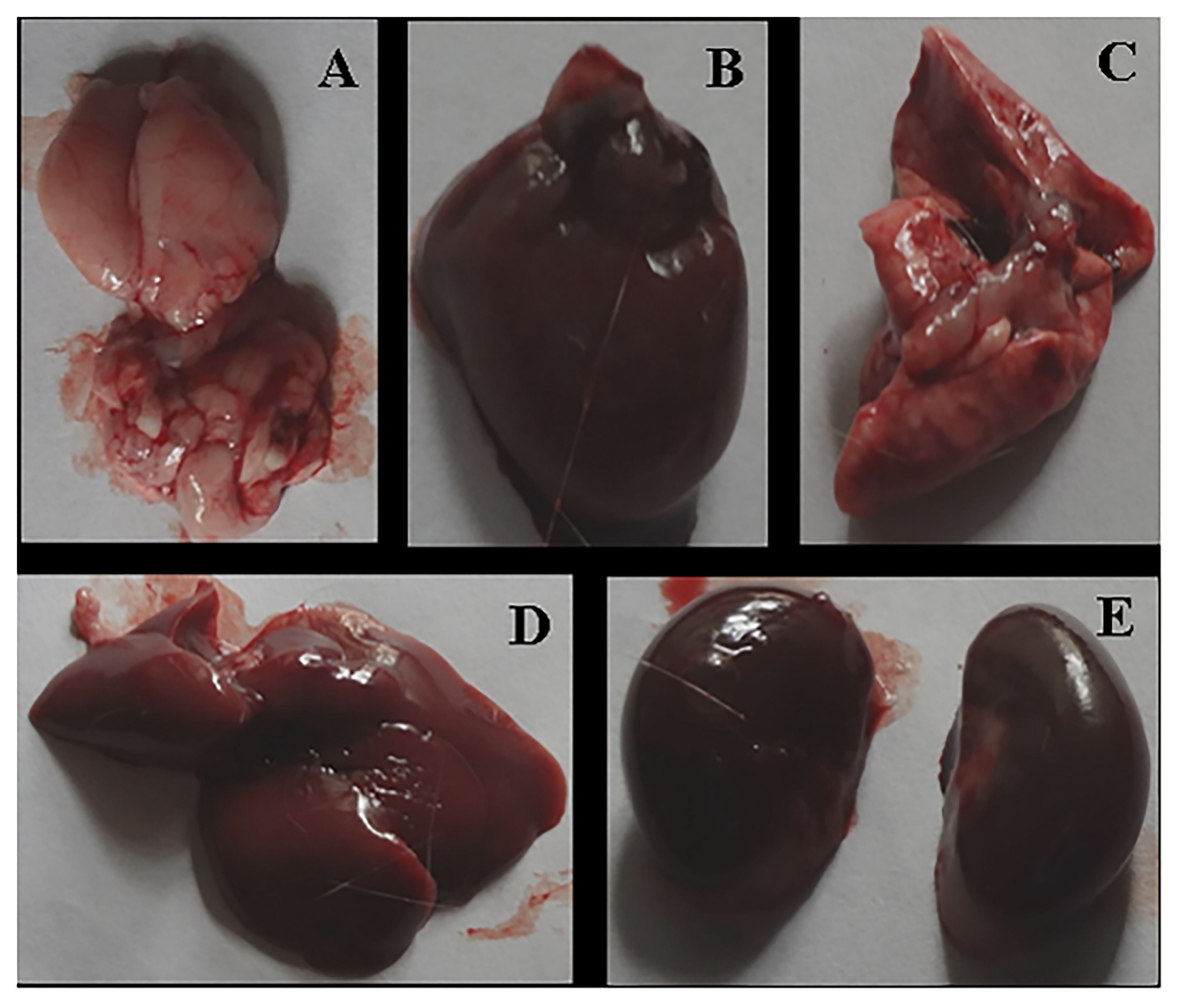
Gross necropsy of albino Wistar rat, organs of untreated (Group F) animal [**(A)** Brain, **(B)** Heart, **(C)** Lung, **(D)** Liver, **(E)** Kidney].

**Figure 7:**
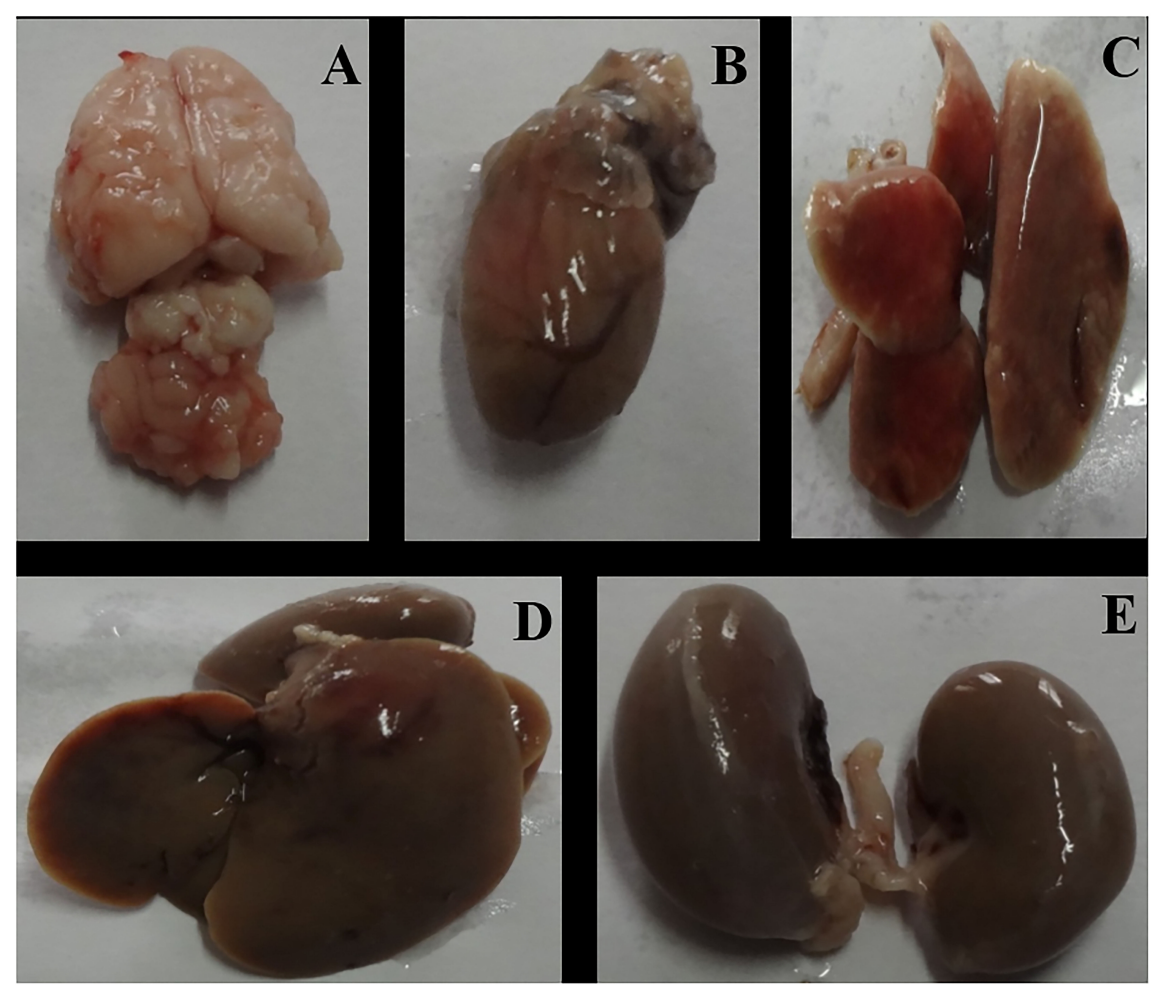
Gross necropsy of albino Wistar rat, organs of experimental (Group C) animal [**(A)** Brain, **(B)** Heart, **(C)** Lung, **(D)** Liver, **(E)** Kidney].

**Figure 8:**
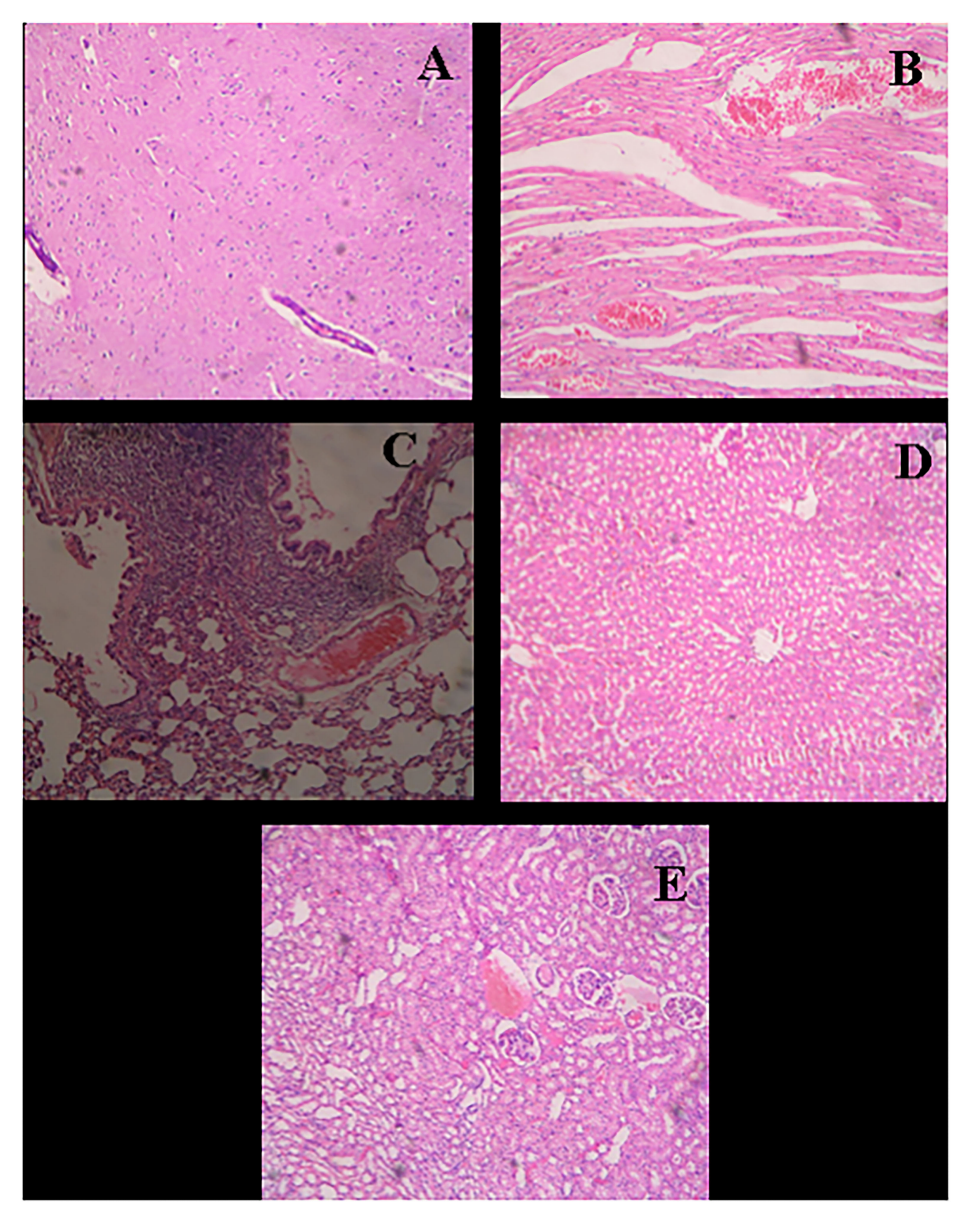
Histological micrograph of organ's tissue after wound-healing study (Group F- Control). **(A)** Section of brain tissue showing normal parenchyma; (**B)** Section of heart tissue showing severe congestion in myocardia; **(C)** Section of lung tissue showing emphysema, round cell infiltration, and imperial bronchiolar congestion; **(D)** Section of liver tissue showing mild congestion and periportal round cell infiltration; **(E)** Section of kidney tissue showing congestion, infiltration, and vacuolar changes in proximal tubule (at a resolution of 40×).

**Figure 9:**
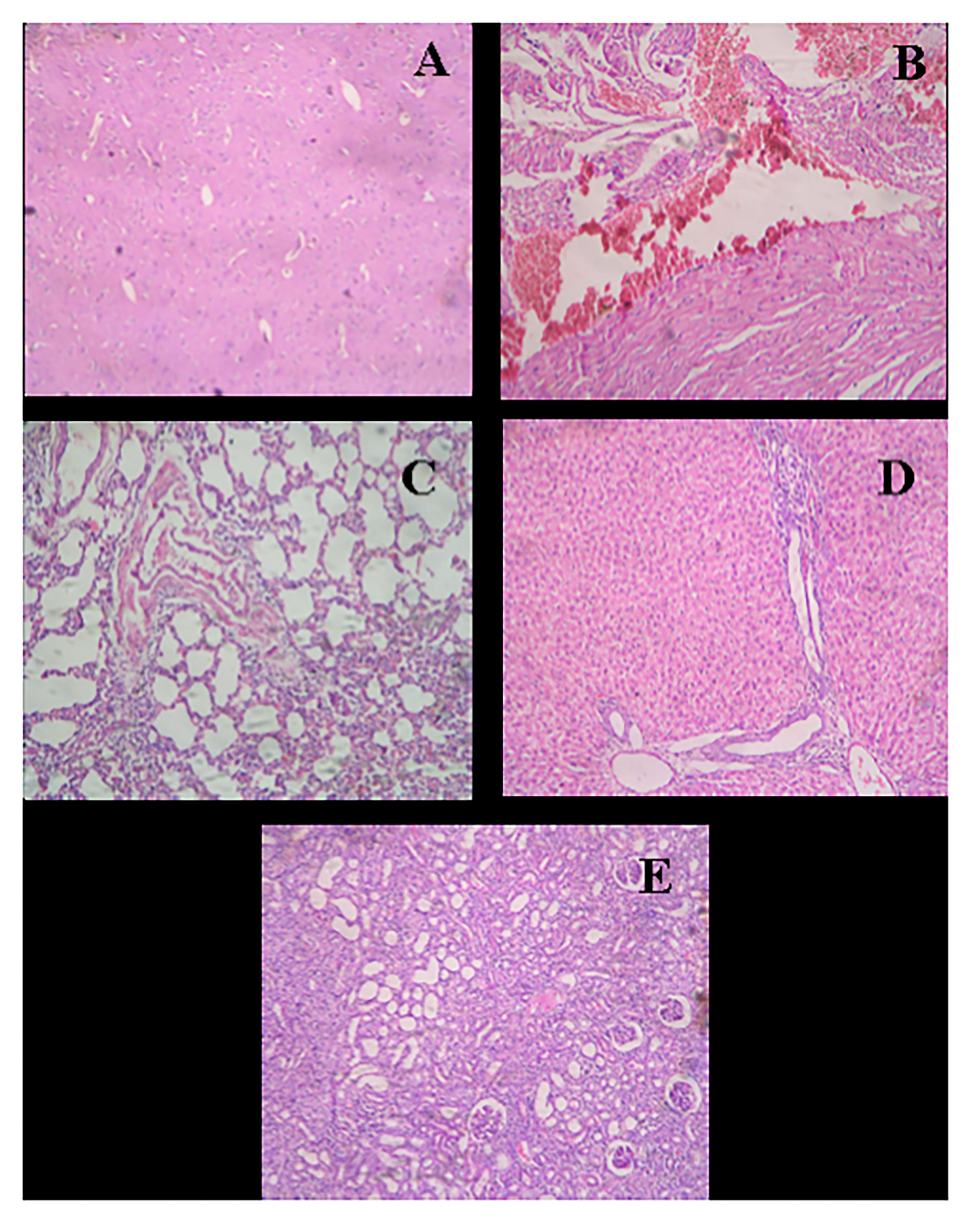
Histological micrograph of organ's tissue after wound-healing study of AgNPs at a concentration of 1 mg g^−1^ (Group C). **(A)** Section of brain tissue showing normal parenchyma; **(B)** Section of heart tissue showing myocardial hemorrhage; **(C)** Section of lung tissue showing bronchiolar round side infiltration and mild congestion; **(D)** Section of liver tissue showing granular and vacuolar changes; **(E)** Section of kidney tissue showing congestion, hemorrhage, and vacuolar changes (at a resolution of 40×).

After the complete study of wound healing, gross necropsy and histology of rat organs were made to evaluate the side effect of NPs on organs, such as brain, heart, kidney, lungs, and liver. First, we observed the physical changes in these organs. There were no physical or any remarkable changes in the organs (Figures 6, 7). Subsequently, a histological study was performed on the tissues of all these organs. In a microscopic analysis of brain tissue, normal parenchyma was observed in both control (Figure 8A) and experimental groups (Figure 9A).

In the study of the heart, experimental tissue showed myocardial hemorrhage (Figure 9B). The control group also revealed severe congestion in myocardia (Figure 8B). In the microscopic study of lung emphysema, round cell infiltration and imperial bronchiolar congestion were observed in the control group (Figure 8C), and the experimental group showed bronchiolar round side infiltration and mild congestion (Figure 9C). The histology of the liver revealed mild congestion and periportal round cell infiltration in the control group (Figure 8D), while the experimental group showed granular and vacuolar changes (Figure 9D). The histology of the kidney exhibited congestion, infiltration, and vacuolar changes in proximal tubule in the control group (Figure 8E), whereas the experimental group exhibited congestion, hemorrhage, and vacuolar changes (Figure 9E).

## Discussion

As mentioned earlier, silver is known since ancient times for its medical uses, including wound-healing property (Mannix-Fisher and McLean, 2021). We developed an antimicrobial silver nanogel and evaluated its wound-healing efficacy on *albino Wistar* rats. Here, we have used AgNPs synthesized from *F. oxysporum*. In a similar study by Nayak et al. (2014), AgNPs synthesized by airborne *Aspergillus* sp. were reported to have a potential antimicrobial activity. AgNPs also enhanced the activity of antibiotics, such as gemifloxacin and moxifloxacin, when assessed against selected clinical pathogens. This emphasized the role of AgNPs as a novel remedy substituent to high-dose antibiotics in the coming future (Akbar et al., 2021).

The tensile strength of a wound represents the degree of wound healing, which is stimulated by applied drugs. These findings show a close relationship with the study by Im et al. (2013) who observed *in vivo* wound-healing activity of sulfate-reduced AgNPs (AS-AgNPs) and chondroitin sulfate-reduced AgNPs (CS-AgNPs) in mice (Hortigüela et al., 2016). Fei et al. (2003) also verified the wound-healing property of 15 UMF manuka honey by enhancing wound tensile strength. In another experiment, Bhubhanil et al. (2021) reported guar gum/curcumin-stabilized AgNPs hydrogel with remarkable wound healing and antibacterial activity. Along with the AgNPs, extracts of plants, such as *Cassis alata*, have proved their superior antimicrobial activity against several common bacterial pathogens, including *E. coli, P. vulgaris, P. aeruginosa, Bacillus subtilis, S. aureus*, and *Serratia marcescens* (Nayak et al., 2015). This enhanced antimicrobial activity intends the use of plant extracts for the treatment of common skin infections caused by these pathogens.

It is reported that collagen plays a key role in wound healing, which promotes the healing activity (Mobaraki et al., 2021). In our study, gel with AgNPs having the concentrations of 0.5 and 1 mg g^−1^ was found to be effective for incision wound healing. It indicates that gel with AgNPs at less concentration doesn't show any toxic or adverse effect and promotes the wound healing of animal tissue with regenerating hair follicles.

Our observations showed a resemblance with Bhuvaneswari et al. (2014) who formulated topical ointment containing AgNPs synthesized using *Naringi crenulata* and evaluated *in vivo* excision wound-healing activity on *albino Wistar* rats. Contraction of a wound involves organizing healthy skin surrounding the wound to cover the denuded area. Myofibroblasts are believed to play a key role in the centripetal movement of the wound margin (Chitturi et al., 2015). Since the AgNPs enhance the contraction of wounds, they might have either improved the contractile property of myofibroblasts or enhanced the number of myofibroblasts in the wound area. In addition, the formulated nanogel has accelerated the period of epithelization. Hence, it is proved that AgNPs promote the wound-healing activity efficiently.

Sundaramoorthi et al. (2009) also found that in the burn wound healing, the re-epithelialization started within 14 days in AgNPs-treated animals; however, the control group took 18 days for re-epithelialization. In our study also, pus formation was observed in the control group, which was not found in the experimental group, as AgNPs are strongly antimicrobial, and also serve as an anti-inflammatory agent in the wound-healing process. The results also corroborate with the findings of Ip et al. (2006) who concluded that silver-coated/impregnated dressings possess strong antimicrobial activity against some burn wound pathogens. Sundaramoorthi et al. (2011) also reported the wound contraction property of the AgNP ointment in different concentrations. Animals treated with 20% AgNPs ointment exhibited a significant wound healing from the fourth day onward, as compared to that sulphadiazine ointment-treated group of animals.

For the production of collagen and other organic components of the intracellular matrix of tissues, ascorbic acid plays an important role. Deficiency of it results in abnormal collagen fibers and changes in the intracellular matrix that expresses as cutaneous lesions, poor adhesion of endothelium cells, and decreased tensile strength of fibrous tissue (Xue and Jackson, 2015; Tracy et al., 2016). In our study, AgNPs showed a remarkable wound-healing activity by increasing cellular proliferation and collagen synthesis at the burn wound site. For many years, silver sulfadiazine has been used for the treatment of burns; however, the benefits of pure silver might be less. Recent developments in nanobiotechnology succeeded to fabricate pure silver in the form of NPs. Therefore, we used pure AgNPs formulation to improve burn wound healing as well as effective antimicrobials.

Hendi (2011) found the fast healing and enhanced cosmetic appearance within 15 days in an animal model. In addition, they claimed that AgNPs showed positive effects due to their antimicrobial activity, reduction in wound inflammation, and modulation in some of the liver and kidney functions during skin wound healing. Keleştemur et al. (2012) also reported that topical application of AgNPs-oligonucleotide increases wound-healing activity by promoting increased collagen synthesis and tissue re-modeling without any side effects on the organs of the mouse. Our results showed a close resemblance with the observation reported by Maneewattanapinyo et al. (2011), who studied the acute toxicity of AgNPs, including eye irritation, corrosion, and dermal toxicity with effect on animal organs. They concluded that there was neither any gross lesion nor histopathological change observed in various organs of AgNPs-treated animals.

In our study, distinct changes were not observed in the organs of the experimental animal as compared to control. However, to evaluate whether the AgNPs have any side effects or not on the animal body, there is a need for further studies including, but not limited to, biochemical tests of organs and other parts of the animals.

## Conclusion

It is evident from this study that silver nanogel with different concentrations, i.e., 0.1, 0.5, and 1 mg g^−1^ of AgNPs synthesized from *F. oxysporum* was successfully formulated using Carbopol. The formulated gel was stable and AgNPs were evenly distributed. Silver nanogel exhibited a remarkable antibacterial activity against *E. coli* and *S. aureus*. The *in vivo* study of formulation exhibited an excellent wound-healing activity in incision, excision, and burn wound-healing model. Gel with AgNP at a concentration of 0.5 mg ml^−1^ showed a superior wound-healing effect, whereas in the study of excision wound-healing model, gel with AgNP at a concentration of 0.1 mg g^−1^ revealed a remarkable wound-healing activity. In the study of burn wound healing, 1 mg g^−1^ showed a better healing effect. In addition, nanogel effectively arrests the bacterial growth on the wound surface and offers an improved local environment for scald wound healing.

Histological studies of healed tissue specify that gel with AgNPs at less concentration does not show any toxic or adverse effect and promotes the wound healing of animal tissue with regenerating hair follicles. On the basis of gross necropsy and histology of rat organs, it can be concluded that there are no distinct changes observed in the organs of the experimental animals as compared to the control.

Finally, *in vitro* and *in vivo* results revealed that the silver nanogel is promising for antimicrobial wound dressing with good biocompatibility for all kinds of wound healing.

## Data Availability Statement

The original contributions presented in the study are included in the article/Supplementary Material, further inquiries can be directed to the corresponding authors.

## Ethics Statement

The animal study was reviewed and approved by Institutional Animal Ethics Committee (Vide letter No. GCOPA/IAEC/2012/404, Institutional Animal Ethics Committee Reg. No. 1370/ac/10/CPCSEA).

## Author Contributions

MR conceptualized the study. API was responsible for the synthesis of AgNPs. AG, PI, and PG were responsible for the characterization of AgNPs. SB and SG formulated nanogel. AG, SB, and SG performed studies on rats and prepared the original draft. PG and MR reviewed and edited the manuscript. PG acquired funds for ACP. All authors have read and accepted the published version of the manuscript.

## Funding

The APC was funded by Nicolaus Copernicus University (IDUB). The authors would like to thank the Rajiv Gandhi Science and Technology Commission, Government of Maharashtra, Mumbai, for financial assistance for this research work. MR and PG would like to thank the Polish National Agency for Academic Exchange (NAWA) for financial support under Grant no. PPN/ULM/2019/1/00117/DEC/1 2019-10-02.

## Conflict of Interest

The authors declare that the research was conducted in the absence of any commercial or financial relationships that could be construed as a potential conflict of interest.

## Publisher's Note

All claims expressed in this article are solely those of the authors and do not necessarily represent those of their affiliated organizations, or those of the publisher, the editors and the reviewers. Any product that may be evaluated in this article, or claim that may be made by its manufacturer, is not guaranteed or endorsed by the publisher.

## Supplementary Material

Supplementary Figure 1 TEM micrograph of silver nanogel at the concentrations of **(A)** 0.1 mg g^−1^; **(B)** 0.5 mg g^−1^; and **(C)** 1 mg g^−1^.
