# Supplementary figures and images for: Superior in vivo Wound-Healing Activity of Mycosynthesized Silver Nanogel on Different Wound Models in Rat
Source: Front Microbiol. 2022 Jun 2;13:881404. doi: 10.3389/fmicb.2022.881404 (PMC9202502; doi:10.3389/fmicb.2022.881404)

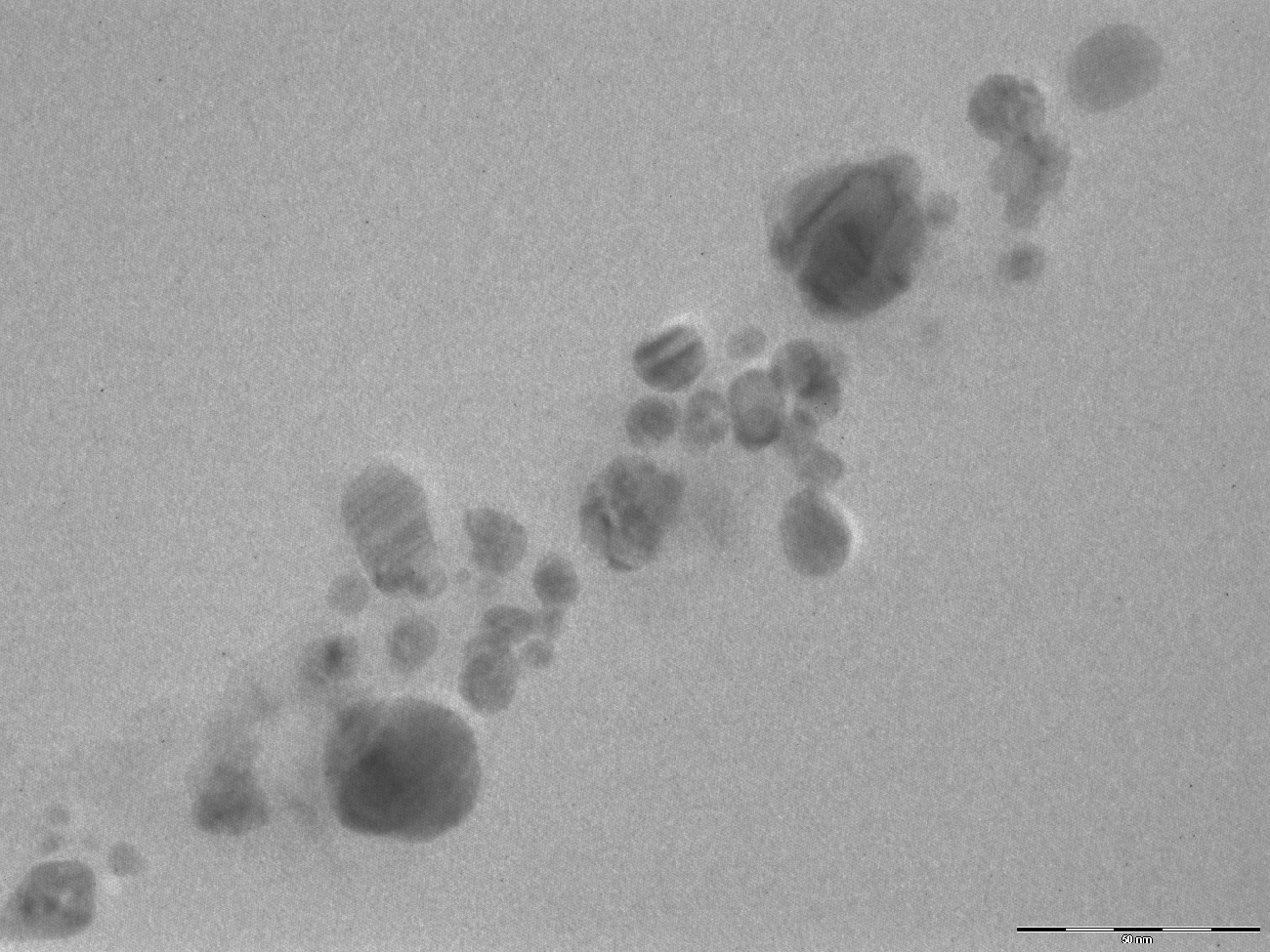

Supplement: Supplementary Figure 1 — TEM micrograph of silver nanogel at the concentrations of (A) 0.1 mg g−1; (B) 0.5 mg g−1; and (C) 1 mg g−1. [file Image_1.TIF]

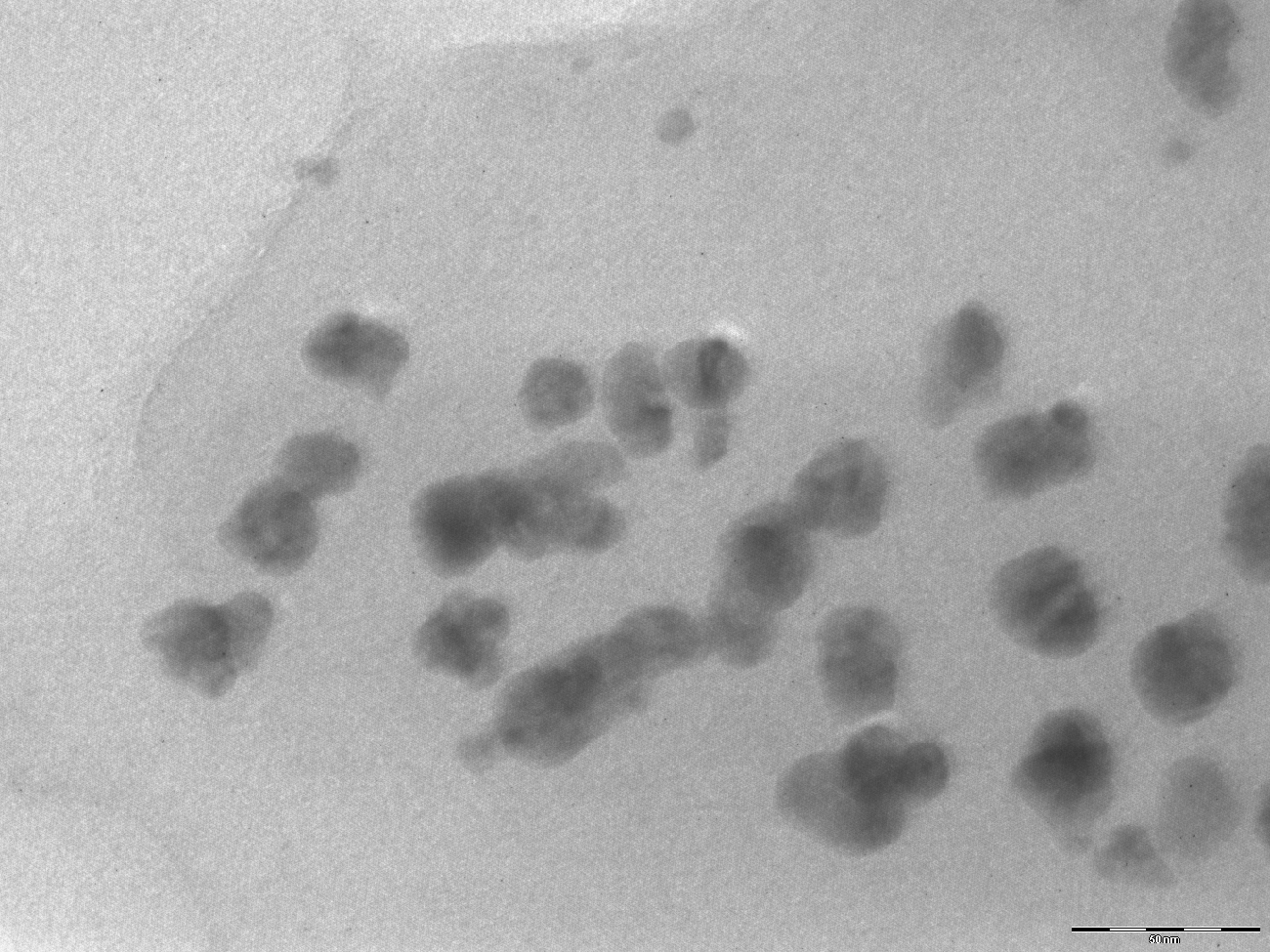

Supplement: Supplementary file 2 [file Image_2.TIF]

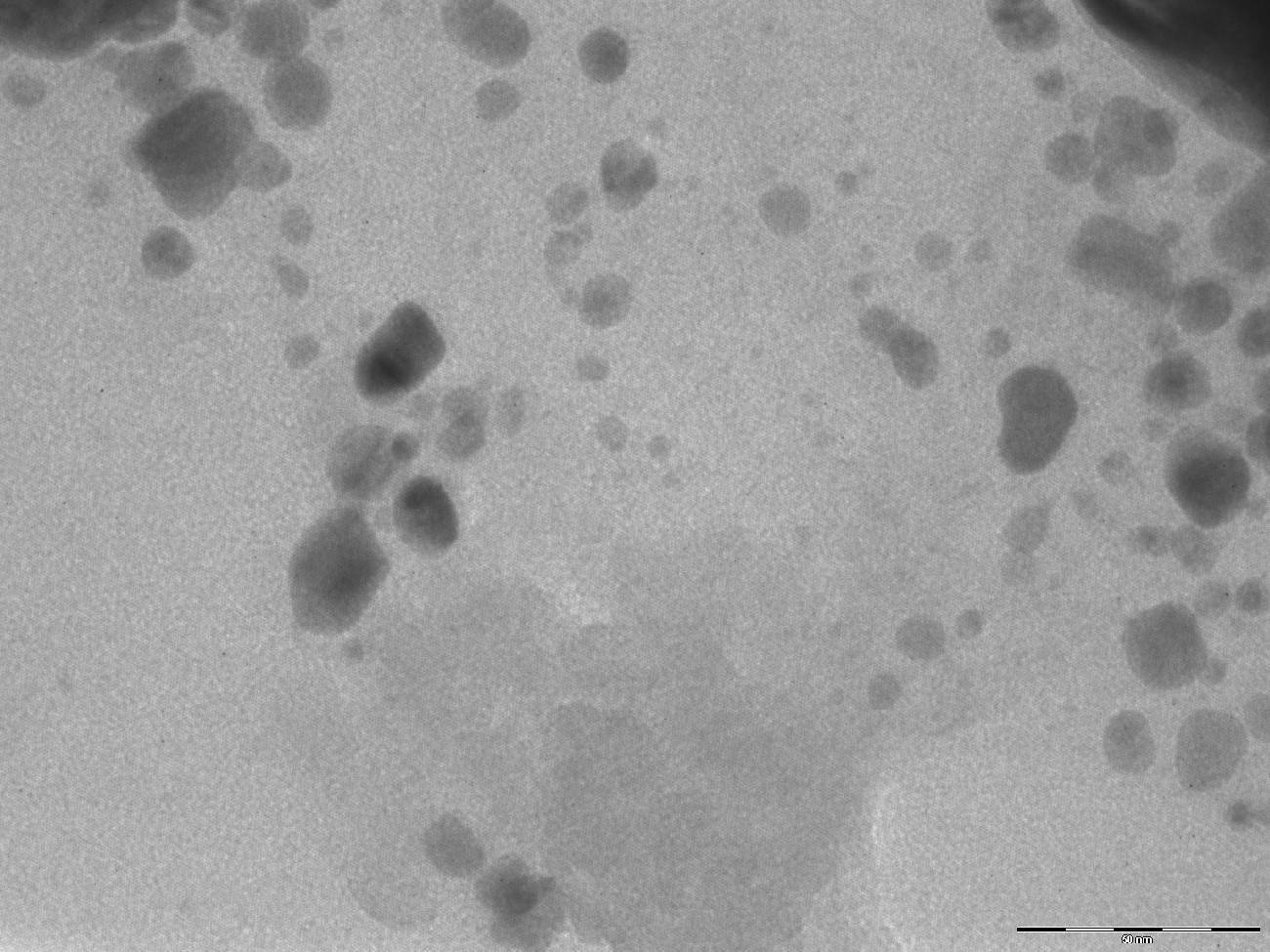

Supplement: Supplementary file 3 [file Image_3.TIF]
